# Whether groups value agreement or dissent depends on the strength of consensus

**DOI:** 10.1371/journal.pone.0334850

**Published:** 2025-12-04

**Authors:** James C. Mellody

**Affiliations:** Center for Information Technology Policy, Princeton University, Princeton, New Jersey, United States of America; City University of Hong Kong, HONG KONG

## Abstract

I investigate the conditions under which groups value agreement versus dissent in collective decision-making processes. I argue that which kind of contribution a group values more will depend on the strength of the consensus. As a consensus evolves from weak to moderate to strong, I predict that groups will prefer agreement, dissent, and then agreement again. These predictions are in line with a multi-phase decision-making process in which groups pursue sequential goals reflected in the evolving consensus: establishing an initial consensus, exploring alternative perspectives, and settling on a final decision. I test these predictions with data from the Reddit community r/AmItheAsshole, in which people make normative judgments of social situations. I find support for the predicted pattern, with one caveat: when the consensus is strong, groups exhibit no preference for agreement or dissent.

## Introduction

Through collective intelligence, the aggregation of inputs from many people can produce solutions and decisions that outperform individual efforts [1–4]. Whereas collective intelligence once emerged only locally, through co-located groups and teams [5], the continued proliferation of the internet has extended the reach of collective intelligence to a wide variety of tasks—including evaluation [6], prediction [7], and innovation [8]—that now benefit from global networks of individuals offering thoughts, ideas, opinions, and judgments.

A large body of work has explored the role of social influence in collective intelligence—mostly investigating how, and under what conditions, social influence either inhibits the diversity of individual judgments and contributes to premature or suboptimal convergence [9,10] or alternatively offers a means of learning that facilitates convergence on optimal judgments from the crowd [11–15].

In addition to the process of making and updating judgments, another key process in collective intelligence is how the collective weights, or values, different kinds of judgments [16]. Much of the research on this process compares different weighting strategies to see which might be the most optimal [17–21]. Given the importance of social influence in the processes of making and updating judgments, it is likely that social influence also plays an important role in the way that collectives value different kinds of judgments in practice.

In this article, I investigate the role of social influence in the process of valuing contributions to the collective. I use the terms ‘group,’ ‘collective,’ or ‘crowd’ to refer to a set of individuals contributing to a single collective decision. I examine how new judgments relate to existing judgments collected by the group and whether this relationship matters for the valuation of these new judgments. More specifically, I ask the following research question: how does the value that a group places on an individual judgment depend on whether the judgment agrees with, and thus bolsters, or disagrees with, and thus questions, the existing group consensus?

There is evidence that groups value both agreement and dissent—as both can facilitate decision making in different ways [22]. Groups value agreement because it fuels convergence on a decision, which is the ultimate goal of many collective intelligence tasks [23–27]. Additionally, groups may value agreement as a means not only of reaching a decision, but also of fostering cohesion, which can veer into groupthink when the pursuit of cohesion incentivizes conformity and crowds out alternative perspectives [28–31]. On the other hand, groups may value dissent—especially when they approach decision making as problem solving—because it represents diversity of thought, which can bolster decision quality [32,33]. Whether a particular group values agreement or dissent may depend on the nature of the task (e.g., where the task falls on the speed-accuracy tradeoff [34]) as well as group norms around consensus and criticality [35].

While particular groups may have a tendency to value agreement or dissent more, it is also possible that, within the course of a single decision-making process, conditions evolve such that groups alternate between valuing agreement more and valuing dissent more. Research on group decision making finds that decision making occurs in phases, across which the motivation of the group can shift. Much of this work suggests a multi-phase process, in which the group’s motivation shifts from general concerns around orienting to a problem, to exchanging ideas, and finally to settling on a solution [36–38]. While there is evidence that the specific dynamics of these phases depend on features of the task and the group [39–41], the broader goals (and thus phases) of establishing and orienting to the problem, exploring potential options, and moving toward a decision, are consistent across different decision-making sequences [40]. These broad phases thus offer a theoretical foundation for hypothesizing about how groups may value different kinds of contributions as a decision-making process evolves.

Further research suggests a link between a group’s motivation and how the group values different kinds of contributions, finding that an approaching deadline can induce a “need for closure," in turn leading groups to reject dissenting opinions in favor of opinion uniformity [42–44]. However, prior work has not examined how a group’s valuation of different kinds of contributions may change dynamically across the phases of decision making. In this article, I address this by investigating whether and how the strength of group consensus serves as a social reference point, shaping whether individuals prioritize agreement or dissent. I argue that the strength of the group consensus offers a social reference point enabling individuals in the collective to track the phase of the decision-making process, such that whether a group places greater value on agreement or dissent will depend on the strength of the consensus.

More specifically—and drawing from research on the benefits of agreement and dissent as well as the phases of decision making—I predict a curvilinear relationship between the strength of consensus and the relative value placed on agreement versus dissent. I predict that when the consensus is weak, groups will value agreement more, in line with a goal of strengthening an initial consensus [23,24]; when the consensus is moderate, groups will value dissent more, in line with a desire to explore diverse perspectives and pressure test the emerging consensus [32,33,36]; and when a consensus is strong, groups will once again value agreement more, in line with a need for closure [36,42–44].

The norms literature offers a potential alternative hypothesis, suggesting a monotonic relationship, such that dissenters are penalized more as group consensus strengthens [22,45]. However, because the present study examines how groups value contributions in specific decision-making contexts, rather than how dissenters are evaluated relative to broader societal norms, I predict that such increased punishment of dissenters will not apply here, and that instead the response to dissenters will evolve in a curvilinear fashion relative to the strength of the consensus.

Additionally, I examine whether the predicted pattern varies by the topic of the decision. Doing so offers an initial exploration into whether the pattern varies by features of the task at hand, which research suggests can shift the dynamics of the phases in decision-making processes [39,40]. While I predict that the general pattern should hold across topics, exploring heterogeneity offers additional insight into how these dynamics may vary, and can suggest potential paths forward for further research on the importance of decision context and topic to how groups value different kinds of contributions.

## Setting and data

**Fig 1:**
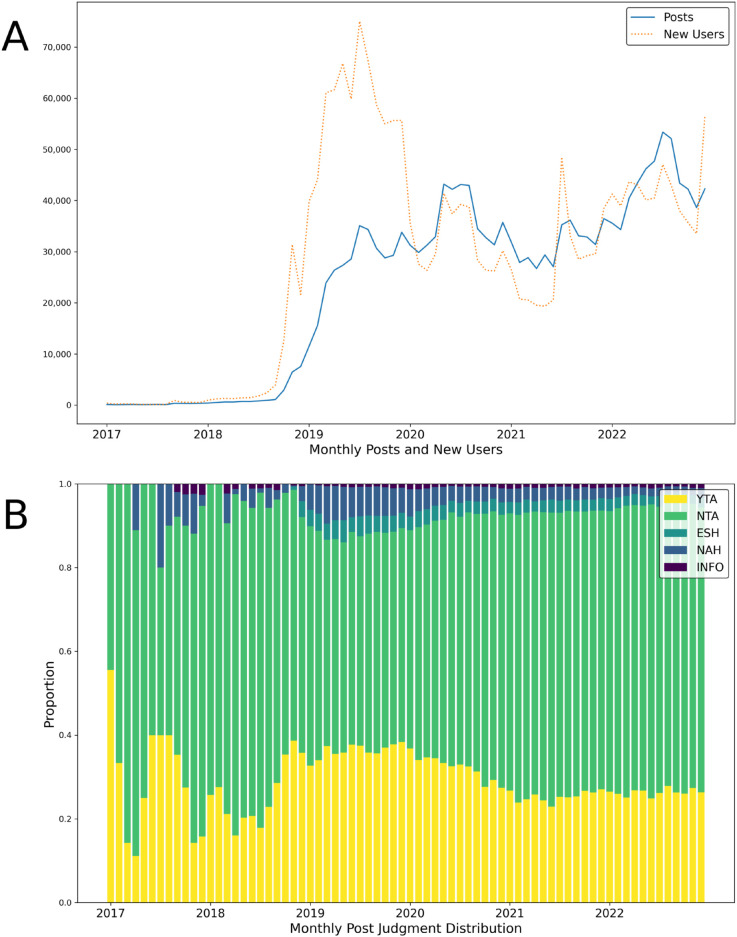
Information about r/AmItheAsshole. Data is from between 2017, before which subreddit activity was limited, and 2022. (A) Number of posts and users each month. The subreddit rapidly increased in popularity in late 2018. (B) Proportion of posts with judgments YTA, NTA, ESH, NAH, and INFO each month (limited to posts with > 14 comments).

I test these predictions in the domain of online evaluations—an increasingly prominent context of collective intelligence [6]. I leverage data from the Reddit community r/AmItheAsshole—a forum that crowd-sources normative evaluations of social situations. In each thread on the forum, the original poster (OP) will post a contentious social situation from their life, and then other people make comments judging who was the “asshole” in the situation. Commenters make one of the following judgments: “you’re the asshole (YTA)” denoting the poster as the asshole, “not the asshole (NTA)” denoting the other party as the asshole, “everyone sucks here (ESH)” denoting all parties as assholes, “no assholes here (NAH)” denoting no parties as assholes, and finally “not enough info (INFO)” to convey that more information is needed to make a judgment. Fig 1 displays descriptive information about the community—including number of posts and users over time, and the distribution of judgments across posts over time.

I focused the analysis on comments made on posts that ultimately received a judgment of either YTA or NTA, because these two judgments were unambiguous. Posts judged as YTA and NTA made up the bulk of posts, as seen in Fig 1. I analyzed only comments that contributed a judgment of YTA or NTA, because these comments had an unambiguous relationship of dissent or agreement relative to the post judgments. I only analyzed top-level comments with judgments, as commenters are supposed to make judgments using top-level comments, according to the subreddit rules.

The rules of the subreddit assign a final post judgment based on the judgment of the comment that received the highest score 18 hours after the post was made. For the purposes of the analyses here, I measured the final judgment in two ways, and then limited the sample to posts for which these two measures agreed (97% of all posts). I did this to ensure that posts in the analyses had an unambiguous judgment, and to capture a more complete picture of the judgment based on the contributions of all in the community, rather than just a single comment. First, I captured the judgment of the single comment that received the highest score. Second, I added up the scores received by comments of each judgment type (“NTA”, “YTA”, “NAH”, “ESH”, or “INFO”) to see which judgment received the highest score (see *S1 Appendix*).

I then tracked the evolving strength of the consensus judgment of the post—a measure I call *Consensus Strength*. I measured this as a function of both the score-weighted proportion of comments at time *t* that expressed the majority judgment (I tested the robustness with an unweighted measure—i.e., proportion of comment counts—and found similar results; see *S5 Appendix*), and the logged number of comments with judgments at time *t* to capture whether the proportion variable reflects many or few comments. In addition to data limitations which exclude the possibility of measuring the evolving score of a single comment, I measured the consensus strength across all comments with judgments in order to capture the evolving consensus of the full group, rather than focusing on the single comment that received the most attention. Doing so captures a more complete picture of the evolving consensus of the group.

In the analysis, I modeled the logged number of upvotes that each comment received—a measure of how the group valued the comment—as a function of the strength of the consensus at the time the comment was made as well as a binary variable indicating whether the focal comment dissented or agreed with the current consensus, along with a number of other control variables. See *Materials and Methods* for more information on the data, measures, and analyses.

## Results

### Main result

**Fig 2:**
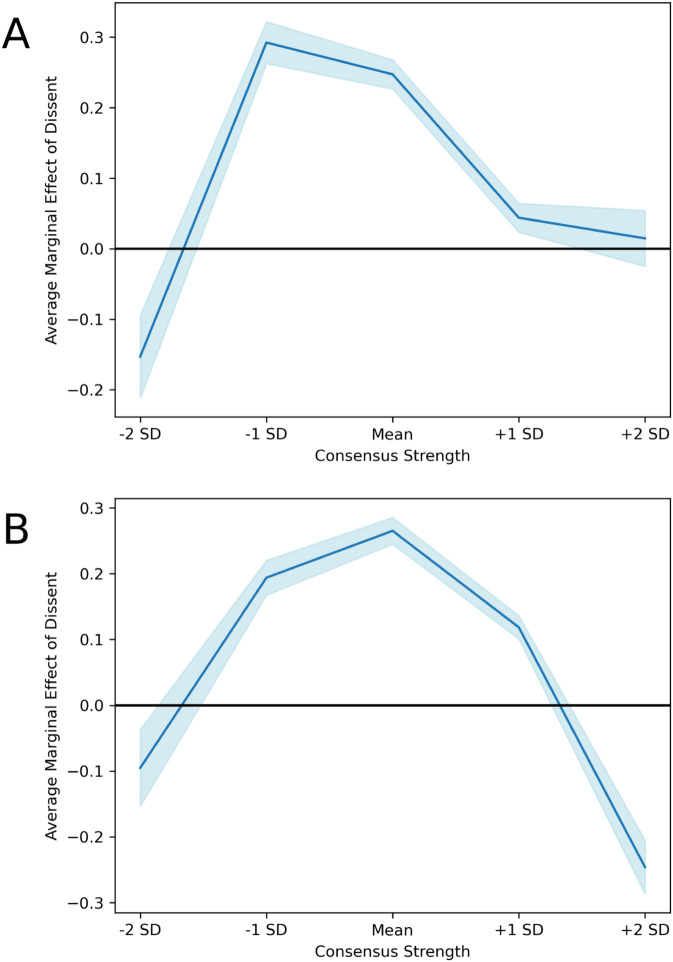
Main result. The main results from A) a model with a cubic term for consensus strength and B) a model with a quadratic term for consensus strength. Results are robust in both models through the second phase, after which they diverge. In the cubic model, there is no preference in this third phase, while the quadratic model shows a shift back to valuing agreement.

The results of the main models offer evidence in support of the prediction that the relative value placed on dissent versus agreement would vary with the strength of consensus, as well as evidence largely in line with the more specific predictions for how this relationship would vary as the consensus evolved. The main result is a curvilinear relationship between the strength of the consensus and the relative value placed on dissent versus agreement. Fig 2 displays the average marginal effect of dissenting with the current consensus on the predicted score (log) of a comment, across different consensus strengths, using a quadratic and then a cubic term to model the evolution of the strength of the consensus. Both models corroborate the first two phases of the core theoretical prediction: a shift from valuing agreement when consensus is weak, to valuing dissent when consensus is moderate. The models diverge in the third phase, in which the quadratic model shows a shift back toward valuing agreement, whereas the cubic model shows a shift toward indifference between dissent and agreement at the highest levels of consensus. This result from the cubic model goes against the prediction for the third phase, which was that groups would prefer agreement in order to close out the decision-making process.

Model diagnostics show identical AIC and BIC values ($1.45\;\times \;10^{7}$), suggesting both models offer a similar fit versus complexity tradeoff, but I will detail the cubic results here, given that the model appears to be picking up an additional statistically significant inflection point in the data. When the consensus strength was very weak (i.e., two standard deviations below the mean), dissenting comments were discounted, receiving an average score that was 14.20% lower (p < 0.001) than the average score of agreeing comments. As the consensus got a bit stronger (i.e., one SD below the mean) the relationship reversed dramatically: dissenting comments received an average score that was 33.97% higher (p < 0.001) than the average score of agreeing comments. At the mean, dissent was still highly favored: dissenting comments received an average score that was 28.06% higher (p < 0.001) than the average score of agreeing comments. As the strength of the consensus further increased (i.e., one SD above the mean), dissent was still slightly favored: dissenting comments received an average score that was 4.49% higher (p < 0.001) than the average score of agreeing comments. Finally, once the consensus was very strong (i.e., two SD above the mean), there was no significant difference in preference for dissenting versus agreeing comments. This main pattern was largely robust across a variety of criteria for filtering the sample (see *S5 Appendix*). See *S4 Appendix* for full regression results from the main models.

I conducted an additional analysis to examine whether the relationship between comment length and comment score differs by agreement with the consensus and the strength of that consensus. Comment length—often a proxy for quality or the depth of explanation—was interacted with consensus strength (cubed) and a binary dissent indicator.

Marginal effects reveal that length matters most when consensus is weakest, and much less when consensus is strong. At a consensus strength 2 SD below the mean, doubling the length of an agreeing comment is associated with a 28.1% increase in score, while a dissenting comment sees a 21.6% increase. At the mean consensus level, these effects drop sharply to 2.0% for agreeing comments and 3.7% for dissenting comments. Once consensus reaches 2 SD above the mean, the boost from doubling length is <1% for agreeing comments and statistically negligible for dissenting comments.

These results suggest that explanations and elaboration play their largest role when consensus is not yet firmly established—possibly because they help sway opinion during this formative stage. As consensus solidifies, such persuasive efforts have diminishing returns. Notably, the pattern also shifts across the consensus trajectory: length appears to matter more first for agreeing comments, then for dissenting comments, before becoming largely irrelevant in the final phase.

### Regression discontinuity in time

To investigate the causal relationship between the strength of consensus and how agreement and dissent are valued, I conducted a regression discontinuity in time (RDiT) analysis [46]. This is a regression discontinuity design in which time operates as the running variable. The goal of this analysis was to see if the kind of contributions the group values would shift after a shock to the strength of the consensus. I used instances when the original poster (OP) of a post made a comment on their own post as a shock to the strength of the consensus. The logic is that the OP commenting on their own post provides new information, painting a more complete picture of the situation and thus lending strength to the consensus. See the *S7 Appendix* for graphical evidence of the jump in the strength of consensus when OP posts a comment. The shift from pre- to post-OP making a comment is not a transition into a fully established consensus, but rather a transition from a first phase of building an initial consensus to a second phase of beginning to explore alternative perspectives. The expectation is that there should be a change in the kind of contribution valued by the group: away from valuing agreement and toward valuing dissent.

**Fig 3:**
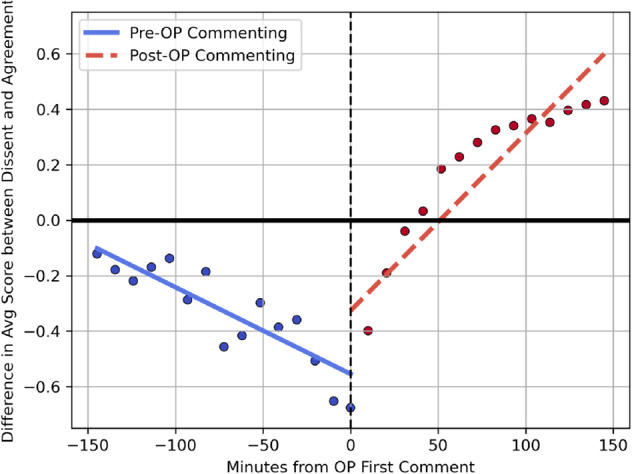
Local discontinuity. This graph shows the local discontinuity in the outcome of interest (difference in average log score between comments that dissented versus agreed with the consensus). This represents the local effect of the original poster’s (OP’s) first comment based on a small bandwidth around the time of treatment: 150 minutes before and after the OP first commented on the post. Outcomes are binned for each 10-minute interval. Before the OP’s first comment, agreement was valued more, whereas after, there was a shift toward valuing dissent.

I used the time difference between the focal comment and the first comment made by the original poster (OP) as the running variable, and I limited the analysis to comments made 150 minutes before and after the OP’s first comment, as RDiT estimates a local treatment effect around the event [46]. Fig 3 shows the discontinuity around the first OP comment: the y-axis displays the mean difference in log score between dissenting and agreeing comments. Before OP made a comment, agreement was valued more, whereas after, there was a shift toward valuing dissent, in line with a move toward exploration. Predictive margins from the RDiT regression offer statistical support in line with these graphical results. Before OP made a comment, dissenting comments received an average log score of 0.74, which was 51% lower (p < 0.001) than the average log score of agreeing comments (1.51). After the OP made a comment, dissenting comments received an average log score of 1.48, which was 3.10% higher (p > 0.001) than the average log score of agreeing comments (1.44). These results offer evidence that a shock to the strength of the consensus can shift the kind of contribution valued by the group, and more specifically, that an increase in the strength of the consensus can shift the group away from valuing agreement and toward valuing dissent.

A potential concern with this RDiT analysis is that the OP’s decision to intervene and comment is dependent on the amount of negativity expressed toward the OP. In other words, the OP may be more likely to comment if the consensus is going against them. To examine whether this pre OP-commenting level of negativity matters for the main effect, I reran the RDiT analysis controlling for the level of pre OP-commenting negativity. To do this, I tracked comments expressing YTA as +1 and comments expressing NTA as −1, weighted by the score of these comments, to produce a score where a larger number reflects greater negativity toward the OP. I also reran the analysis controlling for the strength of the consensus just before the OP commented. For both of these robustness checks, the core finding remains the same: dissent is valued more after the OP comments compared to before, and the strength of this finding remains similar.

### Exploring heterogeneity across topics

**Fig 4:**
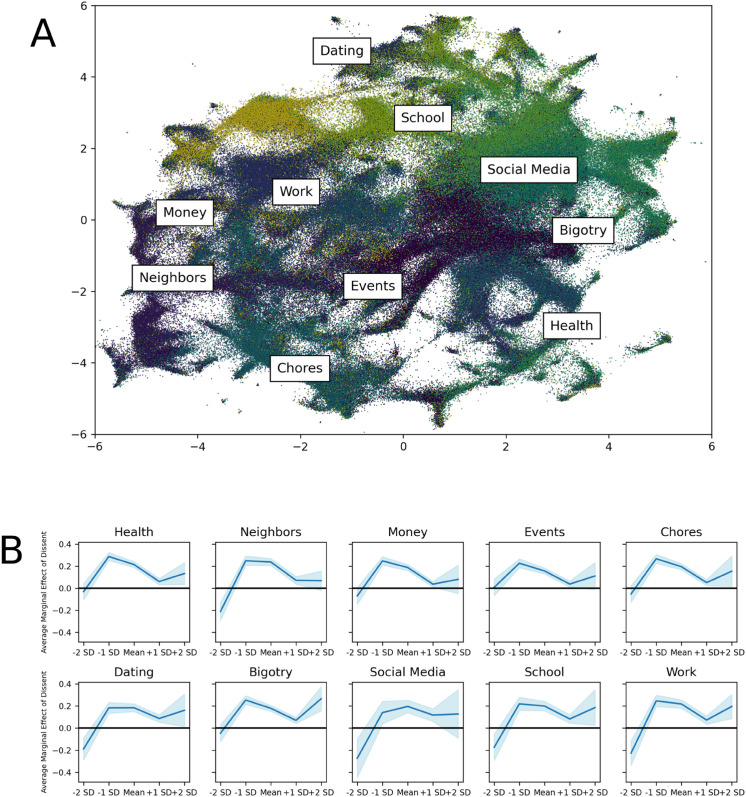
Post topics. (A) 2-dimensional UMAP projection of post embeddings generated through Top2Vec model. (B) Average marginal effects of dissent across topics.

The nature of the data from r/AmItheAsshole enables further testing of the robustness of the main result. Specifically, I can test whether the decision **-**making pattern depends on the topic of the post, or whether the pattern is consistent across topics. To explore these possibilities, I broke the analysis down by post topic. I ran these models with data from the full set of years (i.e., 2013–2022), in order to increase the sample size for each topic. I also leveraged cubic models here, to more flexibly model potential heterogeneity in the patterns. The goal here was to test the generalizability of the main result across different substantive topic areas. To identify the topics of posts, I ran a machine learning model, Top2Vec [47], which makes use of joint document and word embeddings to identify topic vectors. See *Materials and Methods* for more information. The 10 primary topics identified cover a wide range of social situations: health, neighbors, money, events, chores, dating, bigotry, social media, school, and work. Fig 4A displays a two-dimensional UMAP representation of the post embeddings, colored by primary topic.

Fig 4B displays the average marginal effects of dissenting, from the models with the data sub-setted to each of the 10 identified topics. The graphs show a relatively stable pattern across these 10 topics. Variation comes mainly at the extreme ends of the consensus strength spectrum. For some topics (i.e., neighbors, dating, social media, school, and work), agreeing comments were valued more when the consensus was weak—in line with the main pattern, whereas for other topics (i.e., health, money, events, chores, and bigotry), there was not a statistically significant difference between the scores received by dissenting and agreeing comments when the consensus was weak. This suggests that the initial phase of the decision-making process may differ depending on the topic—sometimes the focus of this phase may be valuing agreement to strengthen the consensus, and other times the focus may be on gathering all kinds of contributions.

Similarly, there was variation in how groups valued dissent versus agreement in the final phase: for most topics, in line with the main result, groups exhibited no preference between agreeing and dissenting comments, suggesting that contribution type became less salient once the decision trajectory was established. For a few topics—most significantly bigotry—there was a movement back toward valuing dissent more at the strongest levels of consensus. This finding suggests that the salience of dissenting perspectives in final-phase decision making may depend on the complexity or stakes of the judgment domain, with some contexts requiring ongoing scrutiny even after apparent consensus emergence.

## Discussion

The present study investigated how groups value agreement and dissent throughout the process of making a decision. Leveraging data from the Reddit community r/AmItheAsshole, I found evidence supporting the prediction that the relative value placed on agreement versus dissent would vary with the strength of the consensus. Specifically, I found evidence of a curvilinear relationship between the strength of the consensus and the relative value placed on dissent versus agreement. Groups valued agreement over dissent when a consensus was very weak, placed greater value on dissent when the consensus was moderate, and trended toward not having a preference as the consensus solidified.

This study offers two main contributions. First, this study contributes to our understanding of social influence in collective intelligence. Whereas past work has focused on the role of social influence in how individuals make and update their judgments [9–15], here I focus on the role that social influence plays in how groups value different kinds of contributions. Specifically, I find that the group’s existing consensus operates as a reference point, such that individuals in the group value new contributions based on how these contributions relate to the existing consensus. Together with prior research on social influence in collective intelligence, this helps to paint a more complete picture of exploration and convergence in collective intelligence. Whether, and how, groups explore diverse perspectives and/or converge on particular judgments depends both on how judgments are made and updated, and also on how the group values different kinds of judgments. These processes are interrelated in collective intelligence, as individuals are motivated to take actions that they see receiving recognition [48].

The robustness of main result was further confirmed through additional functional form testing. Cubic and quadratic specifications of consensus strength yielded substantively similar results for the core theoretical prediction—groups initially valued agreement, then shifted to valuing dissent as consensus moved from weak to moderate levels. However, the cubic model revealed additional nuance in the final phase: rather than returning to strong agreement preference, groups with very strong consensus showed indifference between agreement and dissent contributions. This finding suggests that late-stage decision making may be characterized by reduced sensitivity to contribution type, rather than by active agreement-seeking.

Second, this study contributes to research on the phases of group decision making [36–38]. The results offer evidence in support of a three-phase decision-making process, in which groups move from orienting to the problem and building an initial opinion, to evaluating and considering different opinions, and finally to settling on a decision [36]. The present study shows that these different phases have implications for how groups value dissent versus agreement. This matters because decision quality relies on a group’s ability to consider diverse perspectives and avoid converging on suboptimal solutions or ideas.

These findings also connect with and offer insight to theories on the role of disagreement in democratic decision making. Classic political theory, such as Mansbridge’s *Beyond Adversary Democracy* [49], distinguishes between consensus-oriented and adversarial modes of democratic practice, raising the question of when agreement should be sought versus when disagreement should be encouraged. More recent empirical and theoretical work suggests that moderate levels of disagreement can enhance deliberation by fostering problem-solving, prediction, and positive dissensus [50], while too little or too much disagreement can undermine decision quality [51]. The curvilinear pattern observed here—in which groups shift from valuing agreement, to dissent, and then to reduced preference at very strong consensus—aligns with arguments for a dynamic, phase-sensitive approach to democratic deliberation [52]. In this view, the value of dissent is not fixed but depends on where a group is in its decision trajectory, echoing calls for institutions and facilitation strategies that adapt to the deliberative phase [53].

While the specific patterns of valuing agreement and dissent identified in this study may generalize to other online evaluation contexts with similar conditions, it is an open question how far-reaching these patterns are in other settings. The topic-level heterogeneity analyses provide an initial lens on this question. Two noteworthy variations emerged.

First, there was heterogeneity in the initial phase of the decision-making process. Topics that were primarily interpersonal in nature (e.g., neighbors, dating, social media, school, work) followed the main pattern, with groups initially favoring agreement. In contrast, topics that were less directly interpersonal (e.g., health, money, events, chores, bigotry—the last of which is both interpersonal and individual/cognitive) showed no significant early preference. One potential explanation for this divergence draws on structuration theory as applied to small group contexts (e.g., [54–56]). This perspective suggests that decisions about interpersonal matters may follow a logic of “assumed consensus" [55], such that groups are inclined to seek early agreement as a way to reaffirm shared norms and social structure, effectively “grounding” the discussion in a collectively recognized framework before ironing out the details.

Second, in the final phase of consensus evolution, decisions about bigotry diverged from the main pattern. Here, groups tended to value dissent even at high levels of consensus strength, perhaps because of the heightened sensitivity and potential consequences of the topic, leading participants to probe and test agreement more rigorously before closure.

Future research could explore the conditions shaping group preferences for agreement versus dissent at both the early and late stages of consensus building. One such condition may be the group’s entativity—the extent to which members perceive themselves as a unified entity versus a loose collection of individuals [57–59]. In online forums such as Reddit, entativity may be relatively low compared to in-person groups, which could influence receptiveness to different kinds of contributions. Relatedly, group boundaries, their permeability, and the group’s task orientation [35,60,61] may moderate the evolution of preferences for agreement and dissent. Investigating these factors could clarify both the generalizability of the present findings and the mechanisms through which they arise. Another area for future research is examining whether different patterns of alternating between valuing agreement and dissent are more or less optimal for achieving accurate or otherwise effective decisions.

## Materials and methods

### Data and sample

Data was gathered through data dumps provided by Pushshift.io [62]—available from the start of the subreddit in 2013 through the end of 2022, and in accordance with applicable data terms and conditions. For the main analyses, I used matched comments on posts made in 2022—consisting of 6,799,071 comments—because some rules of the subreddit have changed over time and I wanted to capture a period of subreddit stability and maturity, and this is the last full year of Reddit data available. I conducted a robustness check leveraging comments on posts from all years (i.e., 2013-2022), finding largely consistent results (see *S5 Appendix* for this and other robustness checks). Because the goal of the study was to look at the valuation of contributions throughout the course of a decision-making process, I limited the sample to posts which received a minimum of 15 comments with judgments so that each post had a critical mass of judgments. Findings were robust to a different choice of minimum comment threshold. In the main model, I included comments from posts whose main text was deleted by the author or removed by the moderator team, and found the results to be consistent if I excluded these comments. Note that it was a relatively common occurrence for posters to remove their post eventually, but the Reddit AutoModerator bot would repost the original post’s content so that people could keep commenting. In the main analyses, I also removed comments made when the existing consensus was fully balanced (i.e., the proportion variable was equal to 0.5 exactly). This is because the relationship between these comments and the existing consensus was ambiguous. I ran robustness checks including these comments (demarcating them as being in agreement with the existing consensus because a comment made when the consensus is fully balanced effectively establishes a new consensus), and found largely consistent results. There was one important difference to note: when including these comments, at low consensus strength, there was no difference in valuing dissent vs. agreement, possibly because these comments tended to occur when the consensus was weak—because a balanced consensus often occurs early on with only a few comments, meaning the consensus strength is weak. The unclear nature of how these comments relate to the existing consensus likely adds noise at this spectrum of consensus strength, thus why I exclude these comments from the main analysis.

I also limited the analyses to only include comments that received scores equal to or greater than 1. A comment score can take on any integer value. Comments start out with a score of 1, and then can move to a score of 0 and into negative scores if the comment receives more downvotes than upvotes. I limited the sample in this way because the rules of r/AmItheAsshole state that downvoting is reserved not for expressing disapproval of a comment’s judgment, but instead for signaling that a comment is off topic. This means that comments receiving more downvotes than upvotes (i.e., those with a score of 0 or lower) were likely to be irrelevant or off-topic comments, which I wanted to exclude from the analyses.

### Measures

The dependent variable is the score received by a comment. I log-transformed this variable because it is right-skewed. The score was not captured at the exact time the comment was made, but rather after a time lag [62]. Note that the Pushshift project routinely re-retrieved old data from Reddit, such that all comment scores here effectively reflect the final comment score. This is beneficial because a time lag is required for comments to accrue upvotes and downvotes. Given that research shows that attention to social media posts and comments decays exponentially after the time of posting [63], the comment score provides the best available approximation of the comment’s reception close to its original creation time.

In measuring the strength of the consensus, I created a composite measure taking two elements into account. First, in line with measurements of consensus in discrete choice scenarios [64], I measured the strength of the current majority consensus. To measure this, I captured a running tally of the number of comments with the judgment “YTA” and a second running tally of the number of comments with the judgment “NTA.” I then captured the proportion of comments belonging to the majority judgment—meaning a score from (0.5, 1]. This was an unweighted measure of consensus. I also constructed a weighted measure of consensus, as users can upvote a comment if they agree with it, and so the strength of consensus can reflect not just the count of different judgments, but also the number of upvotes that the comments expressing these judgments have received. To construct this weighted measure of consensus, I multiplied each comment by its score, using the sum of these weights for all “YTA” and “NTA” comments to represent the total weighted opinion for each. Note that the weighted and unweighted measures yield similar results, suggesting that the finding is robust to different ways of capturing the consensus strength. I then calculated the proportion of weighted comments contributing to the majority—again a score from (0.5, 1]. I used the weighted measure in the main analysis, and conducted a robustness check with the unweighted measure, finding consistent results (see *S5 Appendix*). Second, I then multiplied the weighted or unweighted proportion variable by the logged number of other comments made at the time of the focal comment. The goal here was to contextualize the proportion variable with information on the volume of comments contributing to that proportion. See the *S2 Appendix* for more information on these measures.

I also measured whether a comment dissented or agreed with the current consensus: *Consensus Dissent*. This is a binary variable—0 if the comment agreed with the consensus, and 1 if it dissented. To capture whether a comment dissented or agreed with the consensus, I measured the judgment of the comment and whether that judgment agreed with the judgment that was currently in the majority under the current consensus. So, for example, if “YTA” currently had a 65% majority, and the focal comment expressed “YTA,” then it was assigned a 0 for this variable, denoting agreement. If the focal comment expressed “NTA," then it was assigned a 1, denoting dissent.

I included several control variables that likely impacted the score received by a comment. I included the length of the comment based on the number of characters: *Comment Length (ln)*, the time in minutes since the original post: *Minutes Since Post (ln)*, the number of other comments in the thread at the time of the focal comment (as a measure of competition for attention)—I added 1 to this variable before taking the log: *Comment Competition (ln)*, the score of the comment author’s other comments made previously in the subreddit (as a measure of author skill at expressing a judgment)—I used STATA’s lnskew0 command to take the log of this variable which ranged from negative to positive values: *Author Score (ln)*, and finally, indicators to capture time-invariant characteristics of the post in which the comment was made, the month of the year in which the comment was made, the hour of the day in which the comment was made, and the day of the week on which the comment was made. See the *S3 Appendix* for descriptive characteristics and correlations for all variables.

### Analyses

I ran linear regressions predicting the score (log) received by each comment. I estimated the following regression equation:


$$y_{i}=\beta_{0}+\beta_{1}D_{i}+\beta_{2}CS_{i}+\beta_{3}CS_{i}^{2}+\beta_{4}CS_{i}^{3}\;\;+\beta_{5}D_{i}CS_{i}+\beta_{6}D_{i}CS_{i}^{2}+\beta_{7}D_{i}CS_{i}^{3}+\delta X_{i}+\alpha_{i}+\alpha_{m}+\alpha_{d}+\alpha_{h}+\epsilon_{i}$$

Where *y*_*i*_ is the logged score of the focal comment, *D*_*i*_ is an indicator for whether the comment dissents with the existing consensus, *CS*_*i*_ is the current strength of the consensus. $\beta_{7}$ is the main quantity of interest: the interaction effect between the dissent variable and the cubed consensus strength term, to account for possible non-linearity. *X*_*i*_ is a vector of covariates and *δ* a vector of accompanying coefficients, $\alpha_{i}$ is a fixed effect for the post in which the focal comment appears, $\alpha_{m}$ is a month fixed effect, $\alpha_{d}$ is a day-of-week fixed effect, $\alpha_{h}$ is an hour-of-day fixed effect, and $\epsilon_{i}$ is the error term. To simplify the interpretation of the interaction effect of interest, I estimated the average marginal effect of dissenting with the consensus, at different consensus strengths, using the STATA margins command.

In order to move toward a causal interpretation of the findings, I took two additional steps. First, I used a coarsened exact matching procedure [65,66] to generate weights for each observation, which I then included in the main regression. This has the dual benefit of improving covariate balance between comparison units (here comments that dissent versus agree with the consensus) and also of operating as a regression pre-processing step that reduces model dependence [67]. I coarsened four variables (i.e., the strength of the consensus, the time since the post in minutes, the score of the author’s other comments in the subreddit, and the length of the comment) into 12 equally sized quantile bins, and a fifth variable (i.e., the number of other comments) into 25 equally sized quantile bins. This process generated weights for each observation, based on how many times each control observation (i.e., agreeing comment) was used as a match for a treated observation (i.e., dissenting comment). I included these weights in the main regression. See *S6 Appendix* for balance table with pre- and post-matching covariate balance, which shows a significant improvement from matching.

Second, I conducted a regression discontinuity in time analysis [46]. I estimated the following equation:


$$y_{i}=\beta_{0}+\beta_{1}D_{i}+\beta_{2}OP_{i}+\beta_{3}D_{i}OP_{i}+\gamma f(TOP_{i})+\delta X_{i}+\alpha_{i}+\alpha_{y}+\alpha_{m}+\alpha_{d}+\alpha_{h}+\epsilon_{i}$$

Where *y*_*i*_ is the logged score of the comment, *D*_*i*_ is an indicator for whether the comment dissents with the existing consensus, *OP*_*i*_ is an indicator for whether the original poster (OP) has posted a comment yet or not, and *TOP*_*i*_ is the running variable (i.e., minutes from first OP comment). Identification comes from the assumption that the potentially endogenous relationship between $\epsilon_{i}$ and the running variable *TOP*_*i*_ is captured by the flexible function f(.) [68]. To estimate this, I included in the model a three-way interaction between *TOP*_*i*_, *D*_*i*_, and *OP*_*i*_, as well as the two-way interactions between *TOP*_*i*_ and each of these other two variables. *X*_*i*_ is a vector of covariates and *δ* a vector of accompanying coefficients, $\alpha_{i}$ is a fixed effect for the post in which the focal comment appears, $\alpha_{y}$ is a year fixed effect, $\alpha_{m}$ is a month fixed effect, $\alpha_{d}$ is a day-of-week fixed effect, $\alpha_{h}$ is an hour-of-day fixed effect, and $\epsilon_{i}$ is the error term. To simplify the interpretation of the interaction effect of interest, I estimated the marginal effect of dissenting with the consensus, before and after the OP’s first comment. I used data from all years for this analysis because data in the small time threshold (150 minutes before/after OP commented) is limited in any given year.

For the heterogeneity in topic analyses, I identified post topics using the Top2Vec algorithm [47]. Top2Vec calculates topic vectors using the following steps: 1) generate joint document and word embeddings—I used the Doc2Vec algorithm here, 2) create lower dimensional embeddings with UMAP, 3) identify dense areas—topics—using HDBSCAN, 4) then calculate the centroid of document vectors in each dense area—this is the topic vector for each dense area, 5) locate the n-closest word vectors which comprise the resulting topic words. I then ran a hierarchical topic reduction algorithm, to reduce the number of topics to ten, and assigned a topic to each post based on the best fit topic. I labeled the topics by inspecting representative topic words from each. See the *S8 Appendix* for example representative topic words from each of the ten topics. I then ran ten regressions, subsetting to each of the topics in turn.

## Supporting information

S1 Appendix Information on how judgments were captured from comments. (PDF)

S2 Appendix Measuring consensus strength. (PDF)

S3 Appendix Descriptive statistics and correlations. (PDF)

S4 Appendix Regression table for main results. (PDF)

S5 Appendix Regression tables for robustness checks. (PDF)

S6 Appendix Matching balance table. (PDF)

S7 Appendix RDiT details. (PDF)

S8 Appendix Topic analysis details. (PDF)
