# Supplementary material for: Whether groups value agreement or dissent depends on the strength of consensus
Source: PLoS One. 2025 Dec 4;20(12):e0334850. doi: 10.1371/journal.pone.0334850 (PMC12677769; doi:10.1371/journal.pone.0334850)
Supplement: S1 Appendix — (PDF) [file pone.0334850.s001.pdf]

S1 Appendix: Capturing Judgments Expressed in Comments

In order to capture the judgment expressed in a comment, I searched the comment text for both the set of abbreviations: YTA, NTA, ESH, NAH, and INFO, as well as alternative abbreviations including YWBTA ("You Would Be the Asshole") and YWNBTA ("You Would Not Be the Asshole"). I also searched for full-text equivalents including the following variations: "youre the asshole," "u r the asshole," "ur the asshole," "you would be the asshole," "you'd be the asshole," "not the asshole," "you would not be the asshole," "you'd not be the asshole", "everyone sucks here," "no assholes here," and "not enough info."
