# Supplementary material for: Whether groups value agreement or dissent depends on the strength of consensus
Source: PLoS One. 2025 Dec 4;20(12):e0334850. doi: 10.1371/journal.pone.0334850 (PMC12677769; doi:10.1371/journal.pone.0334850)
Supplement: S2 Appendix — (PDF) [file pone.0334850.s002.pdf]

S2 Appendix: Measuring Consensus Strength

Here I breakdown the consensus strength measure into its two components. I reran the main analysis with each component in turn, and compared the results to the results from the model using the composite measure (i.e., the main analysis). Average marginal effects of dissenting with the consensus from these three models are shown in Fig S2-1 (cubic) and Fig S2-2 (quadratic). First, I ran the analysis using just the proportion variable, representing the score-weighted proportion of comments expressing the majority judgment, ranging from (0.5, 1]. The first graph in Fig S2-1 (or Fig S2-2) shows that dissent is valued when the proportion is low, and then agreement valued when the proportion is high. Next, I ran the analysis using just the variable capturing the number of other comments existing at the time of the focal comment. The results here, shown in the second graph of Fig S2-1 (or Fig S2-2) show a similar, but more exaggerated pattern, relative to the composite results, which are shown in the third graph of Fig S2-1 (or Fig S2-2).

Together, these results point to the benefits of using a composite measure. On its own, the proportion variable does not provide enough information. The results from the proportion variable show that dissent is valued when the proportion is low, and agreement is valued when the proportion is high. The issue with accepting this pattern on its own is that a proportion variable at one extreme or the other could occur with only a small number of comments (representing a consensus that is likely to change as more comments accumulate) or when there are many comments (representing a well-established consensus that is much more robust). It is more informative to contextualize the proportion variable with the number of comments that contribute to the proportion, as this means the consensus strength reflects the evolving number of comments and thus the stage of the decision making. In other words, a "strong" or "weak" consensus depends on both of these components: the first component—the proportion variable—reflects how strong the group majority is, and the second component—the number of comments making up this proportion—reflects how robust (i.e., likely to change) the majority is.

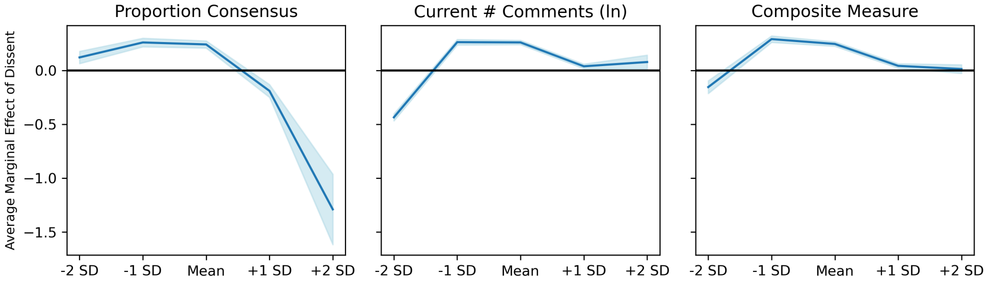

Fig S2-1. Breaking down the consensus strength measure with cubic term. The first graph shows the results using just the proportion variable. The second graph shows the results using just the number of comments (ln) contributing to the proportion. The third graph shows the results using the composite measure.

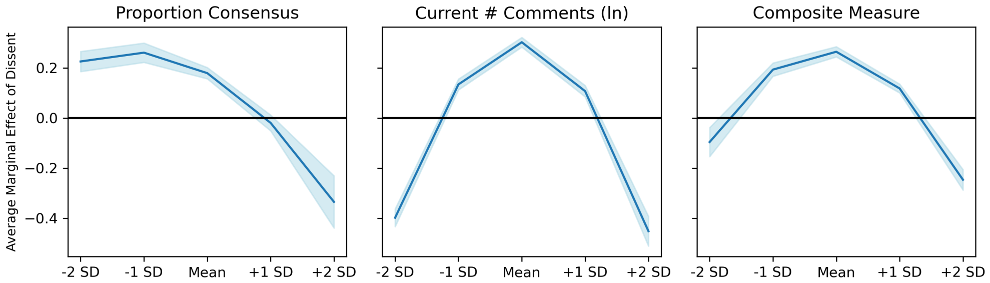

Fig S2-2. Breaking down the consensus strength measure with quadratic term. The first graph shows the results using just the proportion variable. The second graph shows the results using just the number of comments (ln) contributing to the proportion. The third graph shows the results using the composite measure.
