# Supplementary material for: Whether groups value agreement or dissent depends on the strength of consensus
Source: PLoS One. 2025 Dec 4;20(12):e0334850. doi: 10.1371/journal.pone.0334850 (PMC12677769; doi:10.1371/journal.pone.0334850)
Supplement: S3 Appendix — (PDF) [file pone.0334850.s003.pdf]

S3 Appendix: Descriptive Statistics and Correlation Table for Key Variables

Table S3-1. Descriptive statistics.

	Mean	SD	Min	Max
Comment Competition	377.5	591.8	1	8346
Comment Length	285.8	295.1	3	9892
Author Score	15673.0	72886.2	-43816	2846005
Minutes Since Post	782.4	6423.6	0.817	259196
Consensus Strength	4.798	1.675	0.555	9.023
Consensus Dissent	0.0370	0.189	0	1
Observations	6799071			

Table S3-2. Correlation table.

	1	2	3	4	5	6
(1) Comment Competition (ln)	1					
(2) Comment Length (ln)	0.0142	1				
(3) Author Score (ln)	-0.322	0.0434	1			
(4) Mintes Since Post (ln)	0.697	-0.00718	-0.297	1		
(5) Consensus Strength (ln)	0.993	0.0107	-0.318	0.687	1	
(6) Consensus Dissent	0.00395	0.0351	-0.0246	0.0464	-0.0403	1
