# Supplementary material for: Whether groups value agreement or dissent depends on the strength of consensus
Source: PLoS One. 2025 Dec 4;20(12):e0334850. doi: 10.1371/journal.pone.0334850 (PMC12677769; doi:10.1371/journal.pone.0334850)
Supplement: S4 Appendix — (PDF) [file pone.0334850.s004.pdf]

S4 Appendix: Regression Table for Main Result

Table S4. Main regression results.

	(1)	(2)
Consensus Strength	-1.725***	-0.975***
	(0.061)	(0.071)
Consensus Strength^2	0.101***	-0.109***
	(0.002)	(0.010)
Dissent	-0.525***	-1.199***
	(0.056)	(0.079)
Consensus Strength × Dissent	0.374***	1.047***
	(0.024)	(0.058)
Consensus Strength^2× Dissent	-0.044***	-0.226***
	(0.002)	(0.013)
Comment Competition (ln)	0.226***	0.312***
	(0.057)	(0.058)
Min Since Post (ln)	-0.697***	-0.789***
	(0.017)	(0.017)
Min Since Post (ln)^2	0.045***	0.051***
	(0.001)	(0.001)
Author Score (ln)	0.150***	0.150***
	(0.009)	(0.009)
Comment Length (ln)	0.065***	0.064***
	(0.001)	(0.001)
Consensus Strength^3		0.016***
		(0.001)
Consensus Strength^3× Dissent		0.014***
		(0.001)
Constant	5.388***	4.688***
	(0.101)	(0.104)
Post FE	Yes	Yes
Hour FE	Yes	Yes
Day of Week FE	Yes	Yes
Month FE	Yes	Yes
Observations	6,799,071	6,799,071

Note: Standard errors in parentheses are clustered at the post level. Dependent variable is logged comment score. Estimates are from a regression using 2022 data with weights from coarsened exact matching. * $p<0.05$, ** $p<0.01$, *** $p<0.001$ (two-tailed tests). Model 1 contains the quadratic term and model 2 contains the cubic term.
