# Supplementary material for: Whether groups value agreement or dissent depends on the strength of consensus
Source: PLoS One. 2025 Dec 4;20(12):e0334850. doi: 10.1371/journal.pone.0334850 (PMC12677769; doi:10.1371/journal.pone.0334850)
Supplement: S5 Appendix — (PDF) [file pone.0334850.s005.pdf]

S5 Appendix: Robustness Checks for Main Result

I ran a series of robustness checks to test the robustness of the main result to different sample creation strategies. Fig S5-1 displays the average marginal effects of dissenting with the consensus, across these different samples, from the models with the cubic term. These results show that the main pattern is largely robust across these different samples—except for some slight variation in the final phase, in which there is sometimes a leveling off of the preference for dissent, rather than a move toward no preference. Regardless, the curvilinear relationship is consistent across. I conducted all robustness checks on the non-matched sample, because some of the robustness checks included observations which I excluded when doing the matching procedure. Robustness checks included the following: 1) The main sample without matching. 2) The sample including only comments made before the "official" judgment is posted at 18 hours. 3) The sample including posts that meet a minimum comment threshold of 10 comments rather than 15, which was the threshold used in the main analysis. 4) The sample excluding posts that were deleted or removed by either the original author or the moderators. 5) The sample with the unweighted rather than weighted consensus score. 6) The sample including comments that were made when the consensus was balanced. 7) The full sample of years from 2013 - 2022. Fig S5-2 displays the average marginal effects of dissenting with the consensus, across these different samples, from the models with the quadratic term—results are robust again here. Table S5-1 shows the regression results from the cubic models, and Table S5-2 shows the regression results from the quadratic models.

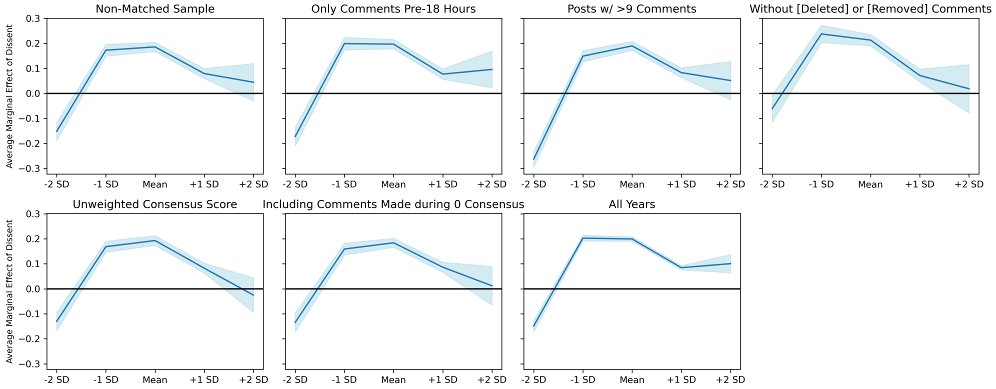

Fig S5-1. Robustness checks with cubic term.

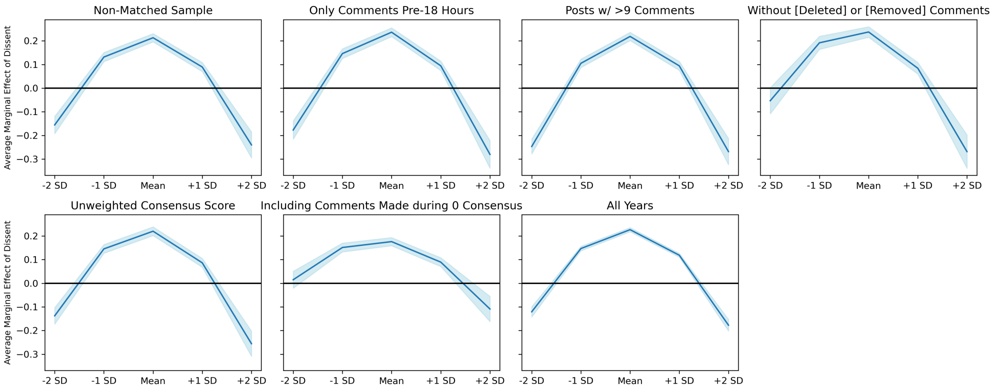

Fig S5-2. Robustness checks with quadratic term.

Table S5-1. Regression results from robustness checks with cubic terms.

	(1) Non-Matched	(2) Pre-18 Hours	(3) >9 Comments	(4) w/o Deleted/Removed Posts	(5) Unweighted Consensus	(6) w/ Balanced Consensus Comments	(7) All Years
Consensus Strength	0.033	0.569***	0.129**	-0.656***		0.163***	-0.256***
	(0.049)	(0.049)	(0.047)	(0.063)		(0.040)	(0.027)
Consensus Strength^2	-0.202***	-0.281***	-0.232***	-0.061***		-0.247***	-0.209***
	(0.010)	(0.010)	(0.010)	(0.013)		(0.008)	(0.006)
Consensus Strength^3	0.020***	0.024***	0.022***	0.011***		0.023***	0.022***
	(0.001)	(0.001)	(0.001)	(0.001)		(0.001)	(0.001)
Dissent	-0.886***	-1.008***	-0.915***	-0.919***	-0.733***	-0.630***	-0.884***
	(0.069)	(0.068)	(0.053)	(0.106)	(0.069)	(0.061)	(0.037)
Consensus Strength × Dissent	0.655***	0.783***	0.668***	0.716***		0.484***	0.733***
	(0.056)	(0.056)	(0.047)	(0.081)		(0.051)	(0.030)
Consensus Strength^2× Dissent	-0.123***	-0.156***	-0.126***	-0.136***		-0.088***	-0.152***
	(0.014)	(0.014)	(0.012)	(0.019)		(0.013)	(0.007)
Consensus Strength^3× Dissent	0.007***	0.009***	0.007***	0.008***		0.005***	0.009***
	(0.001)	(0.001)	(0.001)	(0.001)		(0.001)	(0.001)
Comment Competition (ln)	-0.185***	-0.321***	-0.155***	-0.193***	-0.433***	-0.111**	0.188***
	(0.037)	(0.039)	(0.036)	(0.047)	(0.020)	(0.036)	(0.021)
Min Since Post (ln)	-0.930***	-1.377***	-0.905***	-0.830***	-0.955***	-0.931***	-0.980***
	(0.011)	(0.019)	(0.010)	(0.013)	(0.011)	(0.012)	(0.007)
Min Since Post (ln)^2	0.064***	0.114***	0.062***	0.058***	0.066***	0.064***	0.068***
	(0.001)	(0.002)	(0.001)	(0.001)	(0.001)	(0.001)	(0.001)
Author Score (ln)	0.132***	0.131***	0.129***	0.146***	0.133***	0.136***	0.135***
	(0.003)	(0.003)	(0.002)	(0.005)	(0.003)	(0.003)	(0.002)
Comment Length (ln)	0.084***	0.090***	0.085***	0.081***	0.083***	0.088***	0.085***
	(0.001)	(0.001)	(0.001)	(0.001)	(0.001)	(0.001)	(0.000)
Consensus Strength Raw					0.287***		
					(0.041)		
Consensus Strength Raw^2					-0.199***		
					(0.011)		
Consensus Strength Raw^3					0.020***		
					(0.001)		
Consensus Strength Raw × Dissent					0.545***		
					(0.056)		
Consensus Strength Raw^2× Dissent					-0.098***		
					(0.014)		
Consensus Strength Raw^3× Dissent					0.005***		
					(0.001)		
Constant	4.634***	4.931***	4.351***	5.307***	4.644***	4.277***	25.213***
	(0.066)	(0.146)	(0.062)	(0.081)	(0.066)	(0.063)	(0.568)
Post FE	Yes	Yes	Yes	Yes	Yes	Yes	Yes
Hour FE	Yes	Yes	Yes	Yes	Yes	Yes	Yes
Day of Week FE	Yes	Yes	Yes	Yes	Yes	Yes	Yes
Month FE	Yes	Yes	Yes	Yes	Yes	Yes	Yes
Year FE							Yes
Observations	7,741,416	7,209,646	8,069,059	4,748,336	7,729,811	7,873,728	24404227

Note: Standard errors in parentheses are clustered at the post level. Dependent variable is logged comment score. Estimates are from regressions with each of the following data samples: (1) non-matched sample from 2022, (2) with only comments made before 18 hours after the post, (3) including posts with a minimum comment threshold of 9, (4) without posts that were deleted or removed, (5) using an unweighted consensus strength, (6) including comments made when the consensus was 0, (7) and including all years. * p<0.05, ** p<0.01, *** p<0.001 (two-tailed tests).

Table S5-2. Regression results from robustness checks with quadratic terms.

	(1) Non-Matched	(2) Pre-18 Hours	(3) >9 Comments	(4) w/o Deleted/Removed Posts	(5) Unweighted Consensus	(6) w/ Balanced Consensus Comments	(7) All Years
Consensus Strength	-1.045***	-0.758***	-1.020***	-1.298***		-0.754***	-1.401***
	(0.042)	(0.046)	(0.040)	(0.055)		(0.039)	(0.023)
Consensus Strength^2	0.068***	0.045***	0.062***	0.094***		0.036***	0.075***
	(0.002)	(0.003)	(0.002)	(0.003)		(0.002)	(0.001)
Dissent	-0.599***	-0.652***	-0.647***	-0.512***	-0.561***	-0.165***	-0.485***
	(0.041)	(0.042)	(0.031)	(0.063)	(0.040)	(0.036)	(0.022)
Consensus Strength × Dissent	0.348***	0.389***	0.370***	0.333***		0.161***	0.319***
	(0.019)	(0.020)	(0.016)	(0.027)		(0.017)	(0.011)
Consensus Strength^2× Dissent	-0.037***	-0.043***	-0.040***	-0.037***		-0.019***	-0.036***
	(0.002)	(0.002)	(0.002)	(0.003)		(0.002)	(0.001)
Comment Competition (ln)	-0.216***	-0.318***	-0.188***	-0.222***	-0.446***	-0.129***	0.185***
	(0.036)	(0.039)	(0.035)	(0.046)	(0.020)	(0.036)	(0.021)
Min Since Post (ln)	-0.813***	-1.137***	-0.782***	-0.741***	-0.850***	-0.937***	-0.816***
	(0.012)	(0.020)	(0.011)	(0.016)	(0.012)	(0.010)	(0.007)
Min Since Post (ln)^2	0.057***	0.093***	0.055***	0.052***	0.060***	0.064***	0.055***
	(0.001)	(0.002)	(0.001)	(0.001)	(0.001)	(0.001)	(0.001)
Author Score (ln)	0.138***	0.138***	0.136***	0.149***	0.139***	0.146***	0.141***
	(0.003)	(0.003)	(0.003)	(0.005)	(0.003)	(0.003)	(0.002)
Comment Length (ln)	0.085***	0.091***	0.087***	0.082***	0.085***	0.092***	0.087***
	(0.001)	(0.001)	(0.001)	(0.001)	(0.001)	(0.001)	(0.000)
Consensus Strength Raw					-0.770***		
					(0.027)		
Consensus Strength Raw^2					0.065***		
					(0.002)		
Consensus Strength Raw × Dissent					0.346***		
					(0.020)		
Consensus Strength Raw^2× Dissent					-0.038***		
					(0.002)		
Constant	5.579***	5.835***	5.302***	5.878***	5.555***	4.892***	20.553***
	(0.061)	(0.128)	(0.057)	(0.081)	(0.062)	(0.058)	(0.545)
Post FE	Yes	Yes	Yes	Yes	Yes	Yes	Yes
Hour FE	Yes	Yes	Yes	Yes	Yes	Yes	Yes
Day of Week FE	Yes	Yes	Yes	Yes	Yes	Yes	Yes
Month FE	Yes	Yes	Yes	Yes	Yes	Yes	Yes
Year FE							Yes
Observations	7,741,416	7,209,646	8,069,059	4,748,336	7,729,811	7,873,728	24404227

Note: Standard errors in parentheses are clustered at the post level. Dependent variable is logged comment score. Estimates are from regressions with each of the following data semples: (1) non-matched sample from 2022, (2) with only comments made before 18 hours after the post, (3) including posts with a minimum comment threshold of 9, (4) without posts that were deleted or removed, (5) using an unweighted consensus strength, (6) including comments made when the consensus was 0, (7) and including all years. * p<0.05, ** p<0.01, *** p<0.001 (two-tailed tests).
