# Supplementary material for: Whether groups value agreement or dissent depends on the strength of consensus
Source: PLoS One. 2025 Dec 4;20(12):e0334850. doi: 10.1371/journal.pone.0334850 (PMC12677769; doi:10.1371/journal.pone.0334850)
Supplement: S6 Appendix — (PDF) [file pone.0334850.s006.pdf]

S6 Appendix: Pre and Post Match Balance Table

Table S6. Pre- and post-match balance comparison.

	Pre-Match Agree		Pre-Match Dissent		Post-Match Agree		Post-Match Dissent	
	mean	sd	mean	sd	mean	sd	mean	sd
Comment Competition (ln)	4.838484	1.674438	4.87339	1.56953	4.867974	1.565502	4.87339	1.56953
Comment Length (ln)	5.140801	1.177336	5.360411	1.25418	5.366316	1.217175	5.360411	1.25418
Author Score (ln)	10.86339	.3823207	10.81385	.3166767	10.81659	.3327121	10.81385	.3166767
Minutes Since Post (ln)	5.351071	1.461141	5.710464	1.414893	5.696405	1.415529	5.710464	1.414893
Consensus Strength	4.811491	1.676084	4.454273	1.598241	4.509558	1.569249	4.454273	1.598241
Observations	6547345		251726		6547345		251726	
