# Supplementary material for: Whether groups value agreement or dissent depends on the strength of consensus
Source: PLoS One. 2025 Dec 4;20(12):e0334850. doi: 10.1371/journal.pone.0334850 (PMC12677769; doi:10.1371/journal.pone.0334850)
Supplement: S7 Appendix — (PDF) [file pone.0334850.s007.pdf]

S7 Appendix: Regression Discontinuity in Time

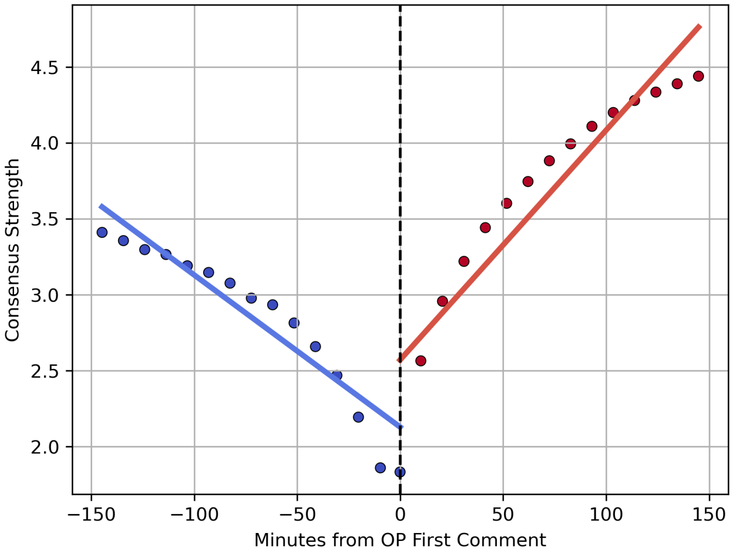

Fig S7. Shift in the strength of the consensus from before to after the original poster makes a comment. This shows an increase in the strength of the consensus after the OP posted.

Table S7. Regression results from RDiT analyses.

	(1)<br>Model 1	(2)<br>Model 2	(3)<br>Model 3
OP Commented	-0.048***	-0.011	-0.018**
	(0.007)	(0.007)	(0.007)
Dissent	-0.575***	-0.562***	-0.568***
	(0.020)	(0.020)	(0.020)
OP Commented × Dissent	0.403***	0.450***	0.452***
	(0.022)	(0.022)	(0.022)
Min Since OP Com	0.007***	0.003***	0.002***
	(0.000)	(0.000)	(0.000)
Min Since OP Com × OP Commented	-0.001***	-0.002***	-0.002***
	(0.000)	(0.000)	(0.000)
Min Since OP Com × Dissent	-0.005***	-0.005***	-0.005***
	(0.000)	(0.000)	(0.000)
Min Since OP Com × OP Commented × Dissent	0.011***	0.010***	0.010***
	(0.000)	(0.000)	(0.000)
Min Since Post (ln)	0.640***	-0.010	-0.217***
	(0.024)	(0.021)	(0.017)
Min Since Post (ln)$\hat{2}$	-0.218***	-0.085***	-0.047***
	(0.003)	(0.003)	(0.002)
Comment Competition (ln)	-0.578***	-0.612***	-0.592***
	(0.014)	(0.013)	(0.013)
Author Score (ln)	0.149***	0.156***	0.158***
	(0.008)	(0.009)	(0.009)
Comment Length (ln)	0.200***	0.200***	0.201***
	(0.001)	(0.001)	(0.001)
Pre OP Consensus Strength		0.398***	
		(0.010)	
Pre OP Com Negativity			-0.000***
			(0.000)
Constant	2.145*	1.938***	2.753***
	(0.887)	(0.112)	(0.110)
Post FE	Yes	Yes	Yes
Hour FE	Yes	Yes	Yes
Day of Week FE	Yes	Yes	Yes
Month FE	Yes	Yes	Yes
Year FE	Yes	Yes	Yes
Observations	3,564,390	3,313,173	3,313,173

Note: Standard errors in parentheses are clustered at the post level. Dependent variable is logged comment score. Estimates are from regression discontinuity in time analyses. Model 1 includes two-way fixed effects for time and the post and represents the main RDiT model. Models 2 and 3 include time fixed effects and a random effect for the post. Model 2 controls for the strength of the consensus pre OP commenting, and model 3 controls for the level of negativity pre OP commenting. * $p<0.05$, ** $p<0.01$, *** $p<0.001$ (two-tailed tests).
