# Supplementary material for: Whether groups value agreement or dissent depends on the strength of consensus
Source: PLoS One. 2025 Dec 4;20(12):e0334850. doi: 10.1371/journal.pone.0334850 (PMC12677769; doi:10.1371/journal.pone.0334850)
Supplement: S8 Appendix — (PDF) [file pone.0334850.s008.pdf]

S8 Appendix: Topic Heterogeneity Analyses

Table S8-1. Representative words from Top2Vec model.

**Health**	**Neighbors**	**Money**	**Events**	**Chores**	**Dating**	**Bigotry**	**Social Media**	**School**	**Work**
doctors	barking	payment	destination	clean	platonic	lgbtq	instagram	grades	employees
overdose	meowing	money	celebration	sink	flirted	racist	snapchat	class	employee
hospitalized	bark	savings	rsvp	moldy	romantically	conservative	insta	scores	manager
alcoholism	dog	purchase	wedding	cleaning	platonically	lgbt	ig	grade	managers
hospital	neighbor	purchases	festivities	cleanliness	flirting	liberal	chats	graded	boss
father	dogs	debt	venue	unwashed	dating	african	deleted	teacher	staffed
complications	neighbors	cash	rsvpd	cleanest	flirty	homophobic	socials	classmates	supervisor
overdosed	leash	debts	plans	slob	situationship	racism	nudes	physics	bosses
opioids	barks	bank	planned	cleaned	dated	ethnically	dms	professor	supervisors
medications	startles	payments	weddings	cleans	romantic	jewish	memes	assignments	understaffed
alcoholic	banging	investments	inviting	dishes	situationships	homosexuality	deleting	math	hires
congestive	yelping	loan	attend	kitchen	flirt	stereotypes	irl	algebra	corporate
opioid	door	mortgage	rsvped	wash	breakup	christians	delete	geometry	employer
pancreatic	noise	frugal	invitation	washed	hooking	bisexual	discord	classes	customers
rehab	growling	loaned	invited	germaphobe	relationship	transgender	whatsapp	classmate	salaried
induced	leashed	monthly	event	dirty	crush	queer	emojis	students	departments
relapsed	roam	earnings	invitations	scrubbing	fwbs	slang	posted	grading	promoted
liver	knocking	investment	celebrations	washing	fwb	gay	twitter	scored	payroll
drug	fenced	funds	rsvping	tidy	friendzoned	trans	profile	assignment	staff
ectopic	woken	cent	invite	laundry	relationships	atheists	posting	academic	department

Table S8-2. Regression results from topic heterogeneity analyses with cubic terms.

	(1)	(2)	(3)	(4)	(5)	(6)	(7)	(8)	(9)	(10)
	Health	Neighbors	Money	Events	Chores	Dating	Bigotry	Social Media	School	Work
Consensus Strength	-0.886***	-0.695***	-0.720***	-0.803***	-0.713***	-0.636***	-0.593***	-0.983***	-0.757***	-0.740***
	(0.087)	(0.086)	(0.074)	(0.092)	(0.080)	(0.108)	(0.074)	(0.222)	(0.115)	(0.095)
Consensus Strength^2	-0.094***	-0.163***	-0.110***	-0.070***	-0.142***	-0.143***	-0.134***	-0.134***	-0.126***	-0.130***
	(0.011)	(0.012)	(0.010)	(0.019)	(0.017)	(0.022)	(0.013)	(0.025)	(0.022)	(0.015)
Consensus Strength^3	0.014***	0.020***	0.015***	0.011***	0.018***	0.017***	0.017***	0.016***	0.017***	0.016***
	(0.001)	(0.001)	(0.001)	(0.002)	(0.002)	(0.002)	(0.001)	(0.002)	(0.002)	(0.001)
Dissent	-1.179***	-1.236***	-0.950***	-0.716***	-1.101***	-0.994***	-0.988***	-0.841**	-1.048***	-1.205***
	(0.135)	(0.139)	(0.132)	(0.143)	(0.156)	(0.168)	(0.140)	(0.268)	(0.192)	(0.168)
Consensus Strength × Dissent	1.006***	1.005***	0.837***	0.646***	0.950***	0.800***	0.903***	0.654**	0.882***	1.045***
	(0.098)	(0.108)	(0.103)	(0.106)	(0.120)	(0.134)	(0.108)	(0.236)	(0.152)	(0.133)
Consensus Strength^2× Dissent	-0.216***	-0.209***	-0.182***	-0.138***	-0.206***	-0.168***	-0.206***	-0.130*	-0.190***	-0.233***
	(0.023)	(0.026)	(0.025)	(0.024)	(0.028)	(0.033)	(0.026)	(0.059)	(0.037)	(0.032)
Consensus Strength^3× Dissent	0.014***	0.013***	0.012***	0.009***	0.013***	0.011***	0.014***	0.008	0.012***	0.016***
	(0.002)	(0.002)	(0.002)	(0.002)	(0.002)	(0.002)	(0.002)	(0.004)	(0.003)	(0.002)
Comment Competition (ln)	0.327***	0.302***	0.226***	0.207**	0.313***	0.345***	0.249***	0.627**	0.256**	0.315***
	(0.077)	(0.075)	(0.065)	(0.063)	(0.061)	(0.089)	(0.061)	(0.207)	(0.088)	(0.084)
Min Since Post (ln)	-1.008***	-0.854***	-0.944***	-1.016***	-0.972***	-0.933***	-0.950***	-0.864***	-0.834***	-0.903***
	(0.022)	(0.020)	(0.021)	(0.023)	(0.025)	(0.033)	(0.023)	(0.038)	(0.030)	(0.029)
Min Since Post (ln)^2	0.071***	0.057***	0.066***	0.073***	0.067***	0.062***	0.063***	0.059***	0.055***	0.061***
	(0.002)	(0.002)	(0.002)	(0.002)	(0.002)	(0.003)	(0.002)	(0.003)	(0.002)	(0.003)
Author Score (ln)	0.144***	0.133***	0.113***	0.130***	0.140***	0.114***	0.134***	0.133***	0.120***	0.139***
	(0.003)	(0.004)	(0.017)	(0.003)	(0.003)	(0.005)	(0.004)	(0.006)	(0.005)	(0.005)
Comment Length (ln)	0.078***	0.089***	0.079***	0.071***	0.082***	0.079***	0.079***	0.090***	0.084***	0.092***
	(0.001)	(0.001)	(0.001)	(0.001)	(0.001)	(0.001)	(0.001)	(0.002)	(0.002)	(0.002)
Constant	-0.225	20.940***	21.386***	34.698***	20.794***	19.630***	0.456	16.860***	14.693***	17.656***
	(0.222)	(1.314)	(0.901)	(1.424)	(1.180)	(1.572)	(0.287)	(1.909)	(1.228)	(1.322)
Post FE	Yes	Yes	Yes	Yes	Yes	Yes	Yes	Yes	Yes	Yes
Hour FE	Yes	Yes	Yes	Yes	Yes	Yes	Yes	Yes	Yes	Yes
Day of Week FE	Yes	Yes	Yes	Yes	Yes	Yes	Yes	Yes	Yes	Yes
Month FE	Yes	Yes	Yes	Yes	Yes	Yes	Yes	Yes	Yes	Yes
Year FE	Yes	Yes	Yes	Yes	Yes	Yes	Yes	Yes	Yes	Yes
Observations	2,850,243	1,739,014	2,149,580	2,372,073	2,071,527	1,055,309	1,852,144	484,813	861,396	975,650

Note: Standard errors in parentheses are clustered at the post level. Dependent variable is logged comment score. Estimates are from regressions with data subsetted to each of the following topics: health, neighbors, money, events, chores, dating, bigotry, social media, school, and work. * p<0.05, ** p<0.01, *** p<0.001 (two-tailed tests).

Table S8-3. Regression results from topic heterogeneity analyses with quadratic terms.

	(1)	(2)	(3)	(4)	(5)	(6)	(7)	(8)	(9)	(10)
	Health	Neighbors	Money	Events	Chores	Dating	Bigotry	Social Media	School	Work
Consensus Strength	-1.669***	-1.770***	-1.538***	-1.467***	-1.699***	-1.564***	-1.543***	-1.820***	-1.690***	-1.589***
	(0.084)	(0.077)	(0.071)	(0.077)	(0.073)	(0.103)	(0.068)	(0.201)	(0.097)	(0.090)
Consensus Strength^2	0.090***	0.104***	0.091***	0.079***	0.098***	0.079***	0.091***	0.073***	0.102***	0.084***
	(0.004)	(0.003)	(0.003)	(0.005)	(0.005)	(0.006)	(0.004)	(0.009)	(0.005)	(0.005)
Dissent	-0.490***	-0.634***	-0.361***	-0.233**	-0.461***	-0.512***	-0.381***	-0.532***	-0.507***	-0.516***
	(0.089)	(0.087)	(0.082)	(0.084)	(0.089)	(0.097)	(0.086)	(0.135)	(0.116)	(0.109)
Consensus Strength × Dissent	0.348***	0.408***	0.277***	0.200***	0.329***	0.316***	0.287***	0.324***	0.334***	0.353***
	(0.039)	(0.042)	(0.037)	(0.036)	(0.040)	(0.045)	(0.039)	(0.068)	(0.054)	(0.054)
Consensus Strength^2× Dissent	-0.040***	-0.046***	-0.033***	-0.023***	-0.039***	-0.034***	-0.034***	-0.035***	-0.037***	-0.040***
	(0.004)	(0.005)	(0.004)	(0.004)	(0.004)	(0.005)	(0.004)	(0.008)	(0.006)	(0.006)
Comment Competition (ln)	0.327***	0.292***	0.216***	0.204**	0.301***	0.353***	0.254***	0.639**	0.253**	0.308***
	(0.075)	(0.074)	(0.065)	(0.063)	(0.060)	(0.087)	(0.060)	(0.200)	(0.088)	(0.081)
Min Since Post (ln)	-0.878***	-0.689***	-0.825***	-0.895***	-0.821***	-0.782***	-0.793***	-0.740***	-0.684***	-0.777***
	(0.024)	(0.020)	(0.022)	(0.030)	(0.030)	(0.036)	(0.024)	(0.044)	(0.029)	(0.031)
Min Since Post (ln)^2	0.062***	0.045***	0.057***	0.065***	0.056***	0.051***	0.052***	0.049***	0.044***	0.052***
	(0.002)	(0.002)	(0.002)	(0.002)	(0.002)	(0.003)	(0.002)	(0.004)	(0.002)	(0.003)
Author Score (ln)	0.148***	0.138***	0.117***	0.132***	0.144***	0.118***	0.138***	0.137***	0.124***	0.143***
	(0.003)	(0.004)	(0.017)	(0.003)	(0.003)	(0.005)	(0.004)	(0.007)	(0.005)	(0.005)
Comment Length (ln)	0.079***	0.091***	0.080***	0.071***	0.083***	0.080***	0.080***	0.091***	0.086***	0.093***
	(0.001)	(0.001)	(0.001)	(0.001)	(0.001)	(0.002)	(0.001)	(0.002)	(0.002)	(0.002)
Constant	1.002***	17.163***	19.522***	32.664***	18.288***	16.567***	2.201***	14.212***	12.052***	15.198***
	(0.233)	(1.240)	(0.912)	(1.494)	(1.194)	(1.597)	(0.300)	(1.974)	(1.244)	(1.355)
Post FE	Yes	Yes	Yes	Yes	Yes	Yes	Yes	Yes	Yes	Yes
Hour FE	Yes	Yes	Yes	Yes	Yes	Yes	Yes	Yes	Yes	Yes
Day of Week FE	Yes	Yes	Yes	Yes	Yes	Yes	Yes	Yes	Yes	Yes
Month FE	Yes	Yes	Yes	Yes	Yes	Yes	Yes	Yes	Yes	Yes
Year FE	Yes	Yes	Yes	Yes	Yes	Yes	Yes	Yes	Yes	Yes
Observations	2,850,243	1,739,014	2,149,580	2,372,073	2,071,527	1,055,309	1,852,144	484,813	861,396	975,650

Note: Standard errors in parentheses are clustered at the post level. Dependent variable is logged comment score. Estimates are from regressions with data subsetted to each of the following topics: health, neighbors, money, events, chores, dating, bigotry, social media, school, and work. * p<0.05, ** p<0.01, *** p<0.001 (two-tailed tests).
